# Barriers and facilitators for self-harm: development and validation of an instrument for health professionals*

**DOI:** 10.1590/1518-8345.7754.4638

**Published:** 2025-08-18

**Authors:** Amanda Sarah Vanzela, Aline Conceição Silva, Laysa Fernanda Silva Pedrollo, Isabela dos Santos Martin, Kelly Graziani Giacchero Vedana

**Affiliations:** 1 Universidade de São Paulo, Escola de Enfermagem de Ribeirão Preto, PAHO/WHO Collaborating Centre for Nursing Research Development, Ribeirão Preto, SP, Brazil; 2 Scholarship holder at the Coordenação de Aperfeiçoamento de Pessoal de Nível Superior (CAPES), Brazil; 3 Universidade de São Paulo, Escola de Enfermagem, São Paulo, SP, Brazil

**Keywords:** Suicide Prevention, Self-Injurious Behavior, Evidence-Based Practice, Validation Study, Health Personnel, Delivery of Health Care

## Abstract

to develop and validate an instrument based on the Theoretical Domains Framework (TDF) to identify barriers and facilitators perceived by health professionals in the prevention of self-harm.

methodological study based on Pasquali’s theoretical framework and the Theoretical Domains Framework. Face and content validations were performed through expert evaluation (n = 13) and construct validity, with health professionals (n = 91). Participants were selected by non-probabilistic convenience sampling. Data were collected on a virtual form and analyzed using the Content Validity Index (CVI), the First-Order Agreement (AC1) coefficient of concordance, and Horn’s exploratory factor analysis.

inter-rater agreement was considered moderate (AC1 = 0.52; SD = 0.04; 95% CI = 0.42 - 0.61; p = ≤ 0.005). After analysis with the target audience, three items were removed (KMO = < 0.6). Factor analysis identified three factors and 11 items, with the internal consistency of the instrument considered high (ω = 0.89). The final version was structured with 11 items on a Likert scale.

the instrument is pioneering and accessible for the Brazilian context, with potential to identify barriers and facilitators self-harm prevention.

## Highlights

**(1)** The instrument identifies barriers and facilitators for the prevention of self-harm. **(2)** The internal consistency of the instrument was considered high. **(3)** The analysis identified the following factors: knowledge and capacity, context and institutional support. **(4)** The instrument developed is easy to use and adaptable to different contexts in Brazil. **(5)** The instrument contributes to studies on the prevention of self-harm.

## Introduction

Suicide is complex, multifactorial, and represents a priority topic on public health agendas worldwide^(1)^. Despite the various prevention strategies that exist, there are still numerous challenges in the development and effective implementation of evidence-based prevention interventions relevant to specific health realities^(1-2)^.

For this study, we used the term self-harm as a type of violence that an individual produces against themselves, including suicidal behavior (suicide attempts and suicide), and nonsuicidal self-injury (NSSI)^(2)^. Every year, thousands of people die by suicide worldwide^(1)^, and in Brazil, in 2021, more than 15 thousand deaths were recorded^(3)^. It is estimated that suicide attempts and NSS may be even more present and numerous, but underreported.

Since this is a multifaceted problem, suicide prevention must be designed and organized based on multilevel and multiprofessional work^(1,4)^. The World Health Organization (WHO) proposes that restricting lethal methods, educating and training health professionals, crisis intervention programs, managing mental disorders, educating media professionals, and post-prevention are effective and necessary strategies for preventing self-harm^(5-6)^.

However, the existence of effective prevention strategies does not presuppose their proper implementation and use in the reality of health professionals. There is a large gap between what is produced by science and what is actually applied in practice^(7-8)^. In this context, it is necessary to understand how evidence-based strategies are and can be effectively implemented in practice^(9)^. Firstly, it is important to identify the context in which a practice will be developed, as well as the factors that influence or do not influence its implementation and results^(7)^.

Researches around the world have attempted to identify the barriers and facilitators for the implementation of strategies for preventing self-harm^(10-11)^. The main barriers pointed by Primary Health Care (PHC) professionals are the lack of training and education among professionals, as well as beliefs and attitudes^(12)^, stigma and judgments^(11,13)^, aspects related to institutional culture^(12)^, and limitations related to financial and human resources^(11,13)^. The main facilitators identified are the training of health professionals, institutional support^(10-11)^, the availability of appropriate and contextualized assessment tools^(10-11)^, public policies that value the rights of victims and access to quality care^(14)^.

It is necessary that implementation studies are developed and based on the specific context in which the strategy will be implemented^(9)^. In Brazil, there are still no instruments that propose to identify barriers and facilitators for the prevention of self-harm from the perspective of health professionals. Studies of this nature still lack a basis in theoretical frameworks that support their understanding.

The Theoretical Domains Framework (TDF) is an integrative theoretical framework developed by psychologists and researchers who are experts in behavior change and the implementation of evidence-based practices. The framework was based on 33 psychological theories and aims to identify the factors that influence the behaviors of health professionals to implement or not (barriers and facilitators) evidence-based practices^(15)^.

The use of this type of tool can help identify barriers and facilitators, contributing to the implementation of evidence-based strategies for preventing self-harm, promoting the improvement of prevention practices and the quality of health work, as well as care for victims and survivors. These findings have the potential to foster discussions on this topic, with a focus on changes in the formulation of public policies for the prevention of self-harm in Brazil. Therefore, the present study aimed to develop and validate an instrument based on the TDF to identify barriers and facilitators perceived by health professionals in the prevention of self-harm.

## Method

### Study design

Methodological study of development and validation of an instrument on barriers and facilitators for the prevention of self-harm, based on the theoretical frameworks of scale development^(16)^ and the TDF^(15)^. The results were described according to PRISMA-COSMIN guidelines^(17)^.

### Setting

The instrument was developed between August and November 2023. Face and content validation took place between January and February 2024 and construct validation between June and July 2024. The entire validation process was carried out through virtual collection, using the Research Electronic Data Capture (REDCap) platform.

### Study steps

The process of constructing the instrument followed theoretical and experimental procedures in order to provide an instrument that could be used in the Brazilian scenario^(16)^. The steps of defining the objectives and target audience of the instrument, and defining the conceptual and operational structures based on the TDF^(15-16)^ were followed. The development of the instrument items was initiated by the authors (AV, AS, LP), with review and discussions with authors IM and KV. In validating the instrument, the steps of face, content and construct validation were followed, as described below.

### Population

The face and content validation of the instrument was carried out with 13 experts, while the construct validation was carried out with 91 health professionals. In both stages, participants were selected using the non-probabilistic convenience sampling technique.

### Selection criteria

The experts were selected using the Lattes platform (an information system on researchers and scientific, technological and innovation institutions in Brazil). The inclusion criteria were: having a background in the health field, a doctorate level and experience (in teaching, research or care) with the topic of self-harm. The data from the experts who met the defined criteria were saved in an editable document, which was used to send invitations during the data collection stage.

To select the target audience, the participation of health professionals working in the Brazilian context was defined, so as to allow the instrument to be valid for people with different levels and professional experiences. The inclusion criteria were to be a Brazilian health professional with professional experience in the country.

### Data collection

Data collection for the face, content, and construct validation stages of the study were carried out on the REDCap platform. When invited to participate in the study, both experts and target audience had access to a form on the platform that contained details of the study, the Free and Informed Consent Form (FICF), a sociodemographic characterization questionnaire, and the instrument for identifying barriers and facilitators for the prevention of self-harm.

For face and content validation, 80 experts were invited via email with information about the study. Of these, 30 showed interest in collaborating and 24 answered the questionnaires. The final sample was composed after excluding questionnaires with incomplete answers or inadequate completion of the instrument’s evaluation items (n = 13).

To select the target audience, health professionals were invited broadly, through posts published on social media (Instagram and Facebook of the research group and researchers responsible for the study). 121 professionals accessed the questionnaire on REDCap. Of these, 98 completed their responses completely, and questionnaires with 15% of missing responses were excluded (n = 7). The sample was then composed by 91 professionals.

Regarding the experts, sociodemographic variables were collected, such as gender, age, race/color, geographic location (region and state), level of education, academic background, length of professional experience, area of activity, experience in teaching, assistance and research, with a focus on self-harm and/or mental health. In addition, the instrument of barriers and facilitators for the prevention of self-harm contained the response options “completely adequate”, “adequate”, “neutral”, “inadequate” and “completely inadequate”. At the end of the instrument, the experts had space to make suggestions for modifying the instrument items.

For the target audience, the same sociodemographic characterization variables mentioned above were collected, as well as the instrument with a response scale of “totally agree”, “agree’,”neutral”, “disagree” and “totally disagree”. In addition, variables related to the ease of completing the instrument, understanding of the response options items, in addition to the relevance of the instrument for clinical practice and suggestions for modifications when pertinent were collected.

### Data processing and analysis

The data obtained were processed in a Microsoft Excel 10 spreadsheet and analyzed using the R program (R core Team, 2023) version 4.3.1 using the Psych package^(18)^. Face and content validity were assessed through descriptive analyses and the Content Validity Index (CVI) with a minimum acceptance or reformulation criterion equal to or greater than 0.80^(19)^. Adequacy was calculated by grouping the options “fully adequate”, “adequate” and “neutral”. The degree of reliability and agreement between the evaluators was assessed using the First-order Agreement Coefficient (AC1). According to this test, the closer to 1.0 the result, the greater the agreement and reliability between experts^(20)^.

Construct validity was assessed considering the dimensionality of the instrument, through exploratory factor analysis (EFA). EFA was used to identify the underlying structure of the instrument items and determine the number of factors that best represent the data. Horn’s Parallel Analysis aimed to define the structure of correlations between items and define a set of dimensions that represent these variables. The Kaiser Meyer Olkin (KMO) was conducted to verify whether the data were suitable for factor analysis. In KMO, the values obtained can vary from zero to one, with values close to zero indicating that the factor analysis is inadequate^(21)^. Values <0.5 are considered unacceptable, values between 0.5 and 0.7 are considered mediocre; values between 0.7 and 0.8 are considered good; values >0.8 are considered excellent; and >0.9 are considered excellent^(22)^.

Bartlett’s sphericity test was also used to assess the adequacy of the factor analysis by assessing the presence of correlation between variables, which is significant when the value between the elements of the main diagonal is equal to one, and absent when the other elements of the matrix are still close to zero^(23)^. The test also assesses the overall significance of all correlations, with p < 0.05 indicating that the matrix is favorable to data factorization^(24)^.

After defining the factors, values equal to or less than 0.40 were excluded from the factor analysis, as they were considered insufficient, i.e., they did not load the identified factor. Finally, McDonald’s ω defined the reliability of the analysis performed.

### Ethical aspects

The study was approved by the Research Ethics Committee of the Ribeirão Preto School of Nursing at the University of São Paulo (CEP/EERP-USP) under opinion number 6,308,906.

## Results

The study was developed in two main stages, namely a) instrument development and b) instrument validation, with the second stage divided between face/content validation and construct validation.

### Instrument development

Initially, statements related to self-harm prevention were developed for each domain (n = 14) and constructs of the TDF (n = 84). The statements were then grouped and synthesized, resulting in 17 items, in addition to two discursive questions to make the response more flexible and to add possible aspects not previously addressed. A five-point Likert scale (Totally agree, Agree, Neutral, Disagree, Totally disagree) was defined as the response scale for the instrument^(25-26)^.

The domains related to the TDF maintained at the end of the construction of the instrument “Barriers and facilitators for self-harm prevention” were: Knowledge (n = 1), Capabilities (n = 1), Identity/professional role (n = 1), Beliefs about capabilities (n = 2), Beliefs about consequences (n = 1), Reinforcement (n = 1), Goals (n = 1), Environmental context and resources (n = 3), Social influences (n = 3), Emotion (n = 2), Behavioral regulation (n = 1).

### Face and content validation with experts

This stage aimed to assess the adequacy, presentation and understanding of the instrument^(16,27)^. The final sample of participants (n = 13) was composed of self-declared female professionals (76.8%), white (76.9%), from the southeast region (69.2%), with training in nursing (92.3%) and doctoral or post-doctoral levels (69.3%). Most participants had professional experience in the field of teaching (84.6%), research (100%) and care (76.9%). In addition, the professionals reported experience in the theme of self-harm (69.2%), and in the field of mental health in primary health care (76.9%). 35.7% had previous experience in studies on the construction and/or validation of instruments.

After analysis by the experts, only items 11, 12 and 15 did not reach the 80% CVI (CVI = 0.69; CVI = 0.77 and CVI = 0.77, respectively) (Table 1), and were improved according to the suggestions received.

In item 11, the experts requested that the term “people I admire” be adapted to “professionals I admire”, to better focus on relationships in the workplace. In item 12, it was suggested that “it causes anxiety, fear…” be replaced by “it causes anxiety, fear…”. In item 15, the experts suggested greater specificity and clarity and the question was changed from “I know which services in the network…” to “I know which services in the health care network and other social devices…”.

In addition, considering the suggestions proposed in the “Suggestions” field of the questionnaire presented to the experts, two other items related to the “Environmental Context and Resources”domain were added”.

**Table 1-:** Reliability and agreement of experts on the instrument “Barriers and facilitators for self-harm prevention” (n = 13). Ribeirão Preto, SP, Brazil, 2024

**Items**	Agreement adequacy N (%)*					CVI**
	**TA** ^†^	**A** ^‡^	**N** ^§^	**I** ^ǁ^	**TI** ^¶^	
Item 1	12 (92.3)	1 (7.7)	-	-	-	1.00
Item 2	10 (76.9)	1 (7.7)	1 (7.7)	1 (7.7)	-	0.85
Item 3	11 (84.6)	1 (7.7)	1 (7.7)	-	-	0.92
Item 4	10 (76.9)	2 (15.4)	1 (7.7)	-	-	0.92
Item 5	10 (76.9)	2 (15.4)	-	1 (7.7)	-	0.92
Item 6	10 (76.9)	1 (7.7)	2 (15.4)	-	-	0.85
Item 7	9 (69.2)	2 (15.4)	1 (7.7)	-	1 (7.7)	0.85
Item 8 ^††^	8 (66.7)	2 (16.7)	1 (8.3)	1 (8.3)	-	0.83
Item 9	12 (92.3)	1 (7.7)	-	-	-	1.00
Item 10	11 (84.6)	-	-	2 (15.4)	-	0.85
Item 11	8 (61.5)	1 (7.7)	2 (15.4)	2 (15.4)	-	**0.69**
Item 12	9 (69.2)	1 (7.7)	1 (7.7)	1 (7.7)	1 (7.7)	**0.77**
Item 13	9 (69.2)	3 (23.1)	-	1 (7.7)	-	0.92
Item 14 ^††^	7 (58.3)	3 (25.0)	1 (8.3)	1 (8.3)	-	0.83
Item 15	9 (69.2)	1 (7.7)	1 (7.7)	1 (7.7)	1 (7.7)	**0.77**
Item 16	10 (76.9)	1 (7.7)	-	2 (15.4)	-	0.85
Item 17	11 (84.6)	-	-	2 (15.4)	-	0.85

*N = Number of participants; ^†^TA = Totally adequate; ^‡^A = Adequate; ^§^N = Neutral; ^ǁ^I = Inadequate; ^¶^TI = Totally inadequate; **CVI = Content validity index; ^††^Missing item

### Construct validation with the target audience

Construct validation aims to assess whether the instrument items actually measure the theoretical concept they are intended to measure. Of the 91 participants, the majority were self-declared female (92.3%), white (74.4%), from the southeast region (85.6%), nursing graduates (71.1%), with a *lato* or *stricto sensu* postgraduate degree (48.4%), and working in the public sector (61.5%). The participants reported not having received prior training on the topic of self-harm (65.9%), having already assisted a victim of self-harm (86.7%), and denied having developed or participated in a self-harm prevention action (56%).

Bartlett’s sphericity test was significant (p < 0.001) and the overall KMO value of the instrument was 0.73, indicating good suitability of the data for factor analysis. Items 5 (beliefs about consequences), 13 (social influences) and 14 (emotions) were excluded because they had KMO < 0.6 (Table 2).

**Table 2-:** Agreement of health professionals in the construct validation of the instrument “Barriers and facilitators for the prevention of self-harm” (n = 91). Ribeirão Preto, SP, Brazil, 2024

**Items**	Agreement N (%)*					**KMO** **
	**CT** ^†^	**C** ^‡^	**N** ^§^	**D** ^ǁ^	**DT** ^¶^	
Item 1	7 (7.8)	35 (38.9)	23 (25.6)	13 (14.4)	12 (13.3)	0.72
Item 2	5 (5.6)	30 (33.3)	14 (15.6)	23 (25.6)	18 (20)	0.77
Item 3	11 (12.1)	23 (25.3)	18 (19.8)	25 (27.5)	14 (15.4)	0.82
Item 4	43 (47.8)	34 (37.8)	8 (8.9)	2 (2.2)	3 (3.3)	0.79
Item 5	70 (76.9)	20 (22)	1 (1.1)	-	-	**0.55**
Item 6	14 (15.4)	13 (14.3)	42 (46.2)	7 (7.7)	15 (16.5)	0.69
Item 7	4 (4.4)	5 (5.5)	15 (16.5)	24 (26.4)	43 (47.3)	0.73
Item 8	3 (3.3)	14 (15.4)	17 (18.7)	24 (26.4)	33 (36.3)	0.71
Item 9	1 (1.1)	20 (22.2)	19 (21.1)	27 (30)	23 (25.6)	0.72
Item 10	5 (5.5)	29 (31.9)	18 (19.8)	20 (22)	19 (20.9)	0.80
Item 11	18 (19.8)	46 (50.5)	21 (23.1)	4 (4.4)	2 (2.2)	0.67
Item 12	8 (8.8)	22 (24.2)	23 (25.3)	20 (22)	18 (19.8)	0.68
Item 13	38 (41.8)	44 (48.4)	-	8 (8.8)	1 (1.1)	**0.57**
Item 14	8 (8.9)	13 (14.4)	26 (28.9)	29 (32.2)	14 (15.6)	**0.54**
Item 15	21 (23.1)	42 (46.2)	14 (15.4)	13 (14.3)	1 (1.1)	0.79
Item 16	6 (6.6)	28 (30.8)	24 (26.4)	22 (24.2)	11 (12.1)	0.74
Item 17	21 (23.1)	30 (33)	15 (16.5)	18 (19.8)	7 (7.7)	0.87

*N = Number of participants; ^†^TC = Totally agree; ^‡^C = Agree; ^§^N = Neutral; ^ǁ^D = Disagree; ^¶^TD = Totally disagree; ^**^KMO = Kaiser-Meyer-Olkin test

**Figure 1-:**
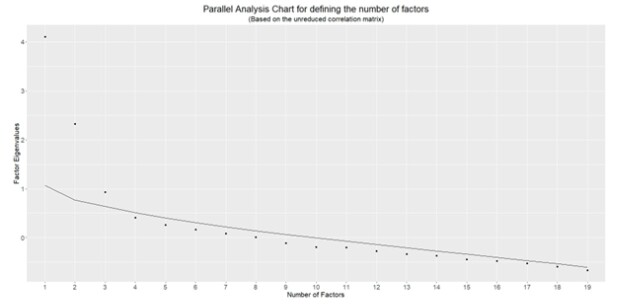
Parallel analysis chart to define the number of factors belonging to the instrument “Barriers and facilitators for the prevention of self-harm”

Horn’s parallel analysis suggested the retention of three factors (knowledge and skills, context and institutional support), considering these factors relevant to the instrument (Figure 1), suggesting the exclusion of items 4 (professional identity), 6 (reinforcement), 11 (emotions), 12 (social influences) and confirming the expected dimensionality.

Table 3 shows the results of the factors (n = 3) and items kept in the final instrument after the oblique rotation test (Oblimin) and extraction method test.

**Table 3-:** Factor loading matrix of the instrument barriers and facilitators for the prevention of self-harm. Ribeirão Preto, SP, Brazil, 2024

Factor	Items	MR1*	MR2	MR3
1	Item 2	0.9161	0.0746	- 0.1050
	Item 1	0.8241	0.1107	- 0.0716
	Item 3	0.7992	- 0.0396	0.0293
	Item 16	0.6929	- 0.0974	0.1575
	Item 15	0.6122	- 0.1319	0.1180
	Item 17	0.5403	- 0.0297	0.2700
			
2	Item 9	0.0515	0.9111	0.0367
	Item 18	-0.1218	0.6045	0.0289
	Item 10	0.0942	0.5640	0.1150
			
3	Item 7	0.0749	- 0.0308	0.7390
	Item 8	- 0.0744	0.2479	0.7110
SS loadings ^†^		3.3752	1.7282	1.3468
Proportion Var ^‡^		0.3068	0.1571	0.1224
Cumulative Var ^§^		0.3068	0.4639	**0.5864**

*Factor; ^†^SS loadings = Sum of squared loadings; ^‡^Proportion Var = Proportion variance; ^§^Cumulative Var = Cumulative variance

The internal consistency of the instrument was assessed using McDonald’s ω and considered good (ω = 0.89). The final items were then renumbered and grouped into three factors that were then named to best represent their domains (Figure 2).

**Figure 2-:** Factors and respective component items of the instrument “Barriers and facilitators for the prevention of self-harm”. Ribeirão Preto, SP, Brazil, 2024

**Factor**	**Items**
**1. Capacity and knowledge**	1. **I am (technically) prepared** to carry out actions to prevent self-harm.
	2. **I know how** to take action to prevent self-harm.
	3. **I feel safe and capable** of taking actions to prevent self-harm.
	4. **I feel safe while caring for** a victim of self-harm in my work environment.
	5. **I look for ways to improve** my performance to contribute to the prevention of self-harm.
	6. **I know which services in the network** (health care and other social devices) I should use to work on preventing self-harm.
**2. Context**	7. **My workplace has physical and material resources** to carry out actions to prevent self-harm.
	8. **The health services available in the network where I work are sufficient to meet the demands** linked to self-harm.
	9. **My workplace has human resources to carry out actions** to prevent self-harm.
**3. Institucional support (reinforcement)**	10. **There are goals/indicators** related to the prevention of self-harm **in my workplace.**
	11. **At my workplace, there is a protocol** for preventing self-harm.

## Discussion

This study developed and validated an instrument for identifying barriers and facilitators for the prevention of self-harm, based on the TDF for use in the Brazilian context. The construction based on the TDF is justified by the scarcity of Brazilian instruments with high theoretical and methodological rigor, developed to assess barriers and facilitators for the prevention of self-harm in the Brazilian context.

The TDF provides a scientifically sound basis for identifying factors that may act as barriers or facilitators in the behavior of professionals, reflecting on their practice. Such identification contributes to the mapping of strategic points, which may favor the implementation of evidence-based practices, or other desirable changes in health services^(28-29)^.

Although mostly used in the development of qualitative instruments, the TDF has been increasingly present in the construction of quantitative questionnaires^(30-32)^. The items developed with a Likert-type response scale can facilitate the collection of quantitative data that are susceptible to generalization, enabling comparisons between different regions of the country. The instrument presented has the advantage of combining a reduced number of items with the scope of TDF constructs. Furthermore, the qualitative aspects of the instrument allow for a greater understanding of factors perceived by participants, providing the opportunity for professionals to expose specific aspects of their experience and place of work. Collecting this information in different contexts can generate relevant insights for implementing interventions that focus on modifiable factors and facilitate the implementation of prevention strategies^(33-34)^.

It is noteworthy that international literature indicates that the main known barriers and facilitators linked to the prevention of self-harm are related to the training of professionals, beliefs and attitudes, institutional culture, financial and human resources, institutional support, assessment tools, public policies and access to quality care^(10-14)^. The instrument developed in this study allows the assessment of these and other factors that may contribute to or hinder suicide prevention in different contexts.

An additional advantage of the instrument is that the statements that make up each item can be partially modified. All items are composed by two parts, one part in bold specifies the characteristic of the TDF domain^(15)^ and another part that mentions “actions to prevent self-harm”, and this part can be adapted to be directed to specific prevention actions, such as crisis management plans, psychoeducation for parents, prevention activities in schools, among others. Thus, the instrument can evaluate preventive actions (in general) or can have all items partially adapted to focus on only one action, such as the crisis management plan. Item 1 “I know how to carry out actions to prevent self-harm” could be adapted to “I know how to carry out crisis management plans” and the other items could be modified in a similar way.

The limitations of the study are related to the fact that the instrument assessments were carried out by professionals who were, predominantly, from the Southeast region of Brazil. Brazil has a vast geographic territory, composed of diversity and singularities, and it is important that the instrument can also identify the barriers and facilitators for the prevention of self-harm in all regions of the country. This study brings relevant contributions to the science of implementation, public policies and improvement of mental health care, as it makes an instrument available free of charge, validated for the Brazilian context. The instrument is in the public domain and can be used by other researchers throughout the country, without the need for prior authorization from the authors (as long as the study is appropriately cited). Open access to the instrument can facilitate researchers from different regions of Brazil to use the instrument in their contexts.

## Conclusion

Based on the results obtained, it is concluded that the instrument presents good adequacy and internal consistency, indicating that the instrument is reliable to measure the proposed constructs. After evaluation by experts on the subject and target audience of the instrument, the final version consisted of 11 questions to be answered on a Likert-type scale and two discursive questions. Thus, the present study resulted in a self-completion instrument, easy to use and subject to adaptations to understand the barriers and facilitators of self-harm for Brazilian contexts. These results indicate the relevance and robustness of the instrument for future research, prevention strategies and health policies.

## References

[1] Organização Pan-Americana da Saúde (2024). LIVE LIFE: An implementation guide for suicide prevention in countries.

[2] World Health Organization (2022). Training manual for surveillance of suicide and self-harm in communities via key informants.

[3] Ministério da Saúde (BR), Secretaria de Vigilância em Saúde e Ambiente (2024). Panorama dos suicídios e lesões autoprovocadas no Brasil de 2010 a 2021. Boletim Epidemiológico.

[4] Hofstra E., Nieuwenhuizen C. van, Bakker M., Özgül D., Elfeddali I., Jong S. J. de (2020). Effectiveness of suicide prevention interventions: a systematic review and meta-analysis. Gen Hosp Psychiatry.

[5] World Health Organization (2021). Comprehensive Mental Health Action Plan 2013–2030.

[6] Mann J. J., Michel C. A., Auerbach R. P. (2021). Improving suicide prevention through evidence-based strategies: a systematic review. Am J Psychiatry.

[7] Wilson P., Kislov R. (2022). Implementation Science.

[8] Mohajerzad H., Martin A., Christ J., Widany S. (2021). Bridging the gap between science and practice: research collaboration and the perception of research findings. Front Psychol.

[9] Bauer M. S., Kirchner J. (2020). Implementation science: What is it and why should I care?. Psychiatry Res.

[10] Davis M., Siegel J., Becker-Haimes E. M., Jager-Hyman S., Beidas R. S., Young J. F. (2023). Identifying common and unique barriers and facilitators to implementing evidence-based practices for suicide prevention across primary care and specialty mental health settings. Arch Suicide Res.

[11] Kasal A., Táborská R., Juríková L., Grabenhofer-Eggerth A., Pichler M., Gruber B. (2023). Facilitators and barriers to implementation of suicide prevention interventions: scoping review. Glob Ment Health (Camb).

[12] Krishnamoorthy S., Mathieu S., Armstrong G., Ross V., Francis J., Reifels L. (2025). Implementation of complex suicide prevention interventions: insights into barriers, facilitators and lessons learned. Arch Suicide Res.

[13] Thangada M. S., Kasoju R. (2024). A systematic review of suicide risk management strategies in primary care settings. Front Psychiatry.

[14] Dantas E. S. O. (2019). Prevenção do suicídio no Brasil: como estamos?. Physis (Rio J).

[15] Cane J., O’Connor D., Michie S. (2012). Validation of the theoretical domains framework for use in behaviour change and implementation research. Implement Sci.

[16] Pasquali L. (1998). Principles of elaboration of psychological scales. Rev Psiquiatr Clin.

[17] Elsman E. B. M., Mokkink L. B., Terwee C. B., Beaton D., Gagnier J. J., Tricco A. C. (2024). Guideline for reporting systematic reviews of outcome measurement instruments (OMIs): PRISMA-COSMIN for OMIs 2024. J Clin Epidemiol.

[18] Revelle W. (2023). Psych: Procedures for Psychological, Psychometric, and Personality Research. R package version 2.3.9.

[19] Polit D., Beck C., Owen S. (2007). Is the CVI an acceptable indicator of content validity? Appraisal and recommendations. Res Nurs Health.

[20] McCray G. (2013). Assessing inter-rater agreement for nominal judgement variables. Language Testing Forum.

[21] Pasquali L. (1999). Instrumentos psicológicos: manual prático de elaboração.

[22] Hutcheson G. D., Sofroniou N. (1999). The multivariate social scientist: introductory statistics using generalized linear models.

[23] Field A. (2005). Discovering Statistics Using SPSS.

[24] Tabachnick B. G., Fidell L. S. (2006). Using multivariate statistics.

[25] Likert R. (1932). A technique for the measurement of attitudes. Arch Psychol.

[26] Dalmoro M., Vieira K. M. (2013). Dilemmas of the type Likert scales construction: does the number of items and the disposition influence results?. RGO.

[27] Echevarria-Guanilo M. E., Gonçalves N., Romanoski P. J. (2019). Psychometric properties of measurement instruments: conceptual basis and evaluation methods - part II. Texto Contexto Enferm.

[28] Abell B., Naicker S., Rodwell D., Donovan T., Tariq A., Baysari M. (2023). Identifying barriers and facilitators to successful implementation of computerized clinical decision support systems in hospitals: a NASSS framework-informed scoping review. Implement Sci.

[29] Correa V. C., Lugo-Agudelo L. H., Aguirre-Acevedo D. C., Contreras J. A. P., Borrero A. M. P., Patiño-Lugo D. F. (2020). Individual, health system, and contextual barriers and facilitators for the implementation of clinical practice guidelines: a systematic metareview. Health Res Policy Syst.

[30] Cunningham S., Jebara T., Stewart D., Smith J., Leslie S. J., Rushworth G. F. (2023). Using the theoretical domains framework to explore behavioural determinants for medication taking in patients following percutaneous coronary intervention. Int J Pharm Pract.

[31] Wong E., Mavondo F., Horvat L., McKinlay L., Fisher J. (2022). Healthcare professionals’ perspective on delivering personalised and holistic care: using the Theoretical Domains Framework. BMC Health Serv Res.

[32] Lynch T., Ryan C., Presseau J., Foster D. E., Huff C., Bennett K. (2024). Development and validation of a theory-based questionnaire examining barriers and facilitators to discontinuing long-term benzodiazepine receptor agonist use. Res Social Adm Pharm.

[33] Spitzner D. J., Meixner C., Liamputtong P. (2023). Handbook of social sciences and global public health.

[34] Silva D. A., Marcolan J. F. (2021). Suicide attempts and suicide in Brazil: an epidemiological analysis. Florence Nightingale J Nurs.

