# Supplementary material for: Barriers and facilitators for self-harm: development and validation of an instrument for health professionals
Source: Rev Lat Am Enfermagem. 2025 Aug 18;33:e4638. doi: 10.1590/1518-8345.7754.4638 (PMC12364452; doi:10.1590/1518-8345.7754.4638)
Supplement: Translation

# Translation: Barreiras e facilitadores para a prevenção da violência autoprovocada: desenvolvimento e validação de instrumento para profissionais de saúde*

**DOI:** 10.1590/1518-8345.7754.4639

**Authors:** Amanda Sarah Vanzela, Aline Conceição Silva, Laysa Fernanda Silva Pedrollo, Isabela dos Santos Martin, Kelly Graziani Giacchero Vedana

**Affiliations:** 1 Universidade de São Paulo, Escola de Enfermagem de Ribeirão Preto, Centro Colaborador da OPAS/OMS para o Desenvolvimento da Pesquisa em Enfermagem, Ribeirão Preto, SP, Brasil; 2 Bolsista da Coordenação de Aperfeiçoamento de Pessoal de Nível Superior (CAPES), Brasil; 3 Universidade de São Paulo, Escola de Enfermagem, São Paulo, SP, Brasil

**Keywords:** Prevenção do Suicídio, Autolesão, Atenção à Saúde Baseada na Evidência, Estudo de Validação, Profissionais da Saúde, Atenção à Saúde

## Abstract

desenvolver e validar um instrumento baseado no *Theoretical Domains Framework* para a identificação de barreiras e facilitadores percebidos por profissionais de saúde na prevenção da violência autoprovocada.

estudo metodológico baseado no referencial teórico de Pasquali e no *Theoretical Domains Framework*. As validações de face e conteúdo foram realizadas mediante avaliação de *experts* (n = 13) e a validade de constructo, com profissionais de saúde (n = 91). Os participantes foram selecionados por amostragem não-probabilística por conveniência. Os dados foram coletados em formulário virtual e analisados a partir do Índice de Validade de Conteúdo (IVC), o coeficiente de concordância *First-Order Agreement* (AC1), e análise fatorial exploratória de Horn.

a concordância entre avaliadores foi considerada moderada (AC1 = 0,52; DP = 0,04; IC 95% = 0,42 - 0,61; p = ≤ 0,005). Após análise com público-alvo, foram removidos três itens (KMO = < 0,6). A análise fatorial identificou três fatores e 11 itens, sendo a consistência interna do instrumento considerada alta (ω = 0,89). A versão final foi estruturada com 11 itens em escala tipo Likert.

o instrumento é pioneiro e acessível para o contexto brasileiro, com potencial na identificação de barreiras e facilitadores para a prevenção da violência autoprovocada.

## Destaques

**(1)** Instrumento identifica barreira e facilitador na prevenção da violência autoprovocada. **(2)** A consistência interna do instrumento foi considerada alta. **(3)** Análise identificou fatores: conhecimentos e capacidade, contexto e apoio institucional. **(4)** Desenvolveu instrumento de fácil uso e adaptável para diversos contextos do Brasil. **(5)** Instrumento colabora com estudos de prevenção da violência autoprovocada.

## Introdução

A violência autoprovocada é complexa, multifatorial e representa um tópico prioritário em agendas de saúde pública no mundo^(1)^. Apesar das diversas estratégias de prevenção existentes, ainda são inúmeros os desafios no desenvolvimento e implementação efetiva de intervenções de prevenção baseadas em evidências relevantes para realidades de saúde específicas^(1-2)^.

A violência autoprovocada é conceituada como um tipo de violência que um indivíduo produz contra si mesmo, incluindo o comportamento suicida (tentativas e suicídio), além das autolesões não suicidas (ALNS)^(2)^. Todos os anos milhares de pessoas morrem por suicídio em todo o mundo^(1)^, sendo que no Brasil, em 2021, mais de 15 mil mortes foram registradas^(3)^. Estima-se que as tentativas de suicídio e as ALNS são ainda mais presentes e numerosas, porém subnotificadas.

Tratando-se de um problema multifacetado, a prevenção da violência autoprovocada deve ser pensada e organizada a partir de um trabalho multinível e multiprofissional^(1,4)^. A Organização Mundial da Saúde (OMS) propõe que a restrição de métodos letais, a educação e treinamento de profissionais de saúde, programas de intervenção de crise, manejo de transtornos mentais, educação de profissionais da mídia e posvenção são estratégias eficazes e necessárias para a prevenção de violência autoprovocada^(5-6)^.

No entanto, a existência de estratégias efetivas para a prevenção não pressupõe a devida implementação e uso das mesmas na realidade dos profissionais de saúde. Existe uma grande lacuna entre o que é produzido pela ciência e o que é realmente aplicado na prática^(7-8)^. Nesse contexto, é necessário compreender como as estratégias baseadas em evidências são e podem ser efetivamente implementadas na prática^(9)^. Sendo assim, primeiramente é importante identificar o contexto em que uma prática será desenvolvida, bem como os fatores que influenciam ou não em sua implementação e resultados^(7)^.

Pesquisas no mundo tentam identificar as barreiras e facilitadores para a implementação de estratégias para a prevenção da violência autoprovocada^(10-11)^. As principais barreiras ligadas à prevenção da violência por profissionais da Atenção Primária à Saúde (APS) são a falta de treinamento e educação de profissionais, crenças e atitudes^(12)^, estigma e julgamentos^(11,13)^, aspectos ligados à cultura institucional^(12)^, bem como limitações ligadas a recursos financeiros e humanos^(11,13)^. Os principais facilitadores identificados são a formação de profissionais de saúde, o apoio institucional^(10-11)^, a disponibilidade de ferramentas de avaliação adequadas e contextualizadas^(10-11)^, políticas públicas que valorizem os direitos das vítimas de violência e o acesso à assistência de qualidade^(14)^.

É necessário que estes estudos de implementação de práticas sejam desenvolvidos e baseados no contexto específico no qual a estratégia será implementada^(9)^. No Brasil, ainda não existem instrumentos que propõem identificar barreiras e facilitadores para a prevenção da violência autoprovocada na perspectiva de profissionais da saúde. Estudos dessa natureza ainda carecem de embasamento em referenciais teóricos que sustentem a compreensão das mesmas.

O Theoretical Domains Framework (TDF) trata-se de uma estrutura teórica integrativa construída por psicólogos e pesquisadores *experts* em mudança do comportamento e implementação de práticas baseadas em evidências. A estrutura foi fundamentada em 33 teorias da psicologia e se propõe a identificar os fatores que influenciam nos comportamentos de profissionais de saúde para implementar ou não (barreiras e facilitadores) práticas baseadas em evidências^(15)^.

A utilização desse tipo de ferramenta pode auxiliar na identificação de barreiras e facilitadores, contribuindo para a implementação de estratégias de prevenção da violência autoprovocada baseadas em evidências, promovendo a melhora de práticas de prevenção e a qualidade do trabalho em saúde, bem como do atendimento às vítimas e sobreviventes. Esses achados apresentam potencial para fomentar discussões sobre essa temática, com foco em mudanças, no que se refere à formulação de políticas públicas de prevenção da violência autoprovocada no Brasil. Sendo assim, o presente estudo teve o objetivo de desenvolver e validar um instrumento baseado no TDF para a identificação de barreiras e facilitadores percebidos por profissionais de saúde na prevenção da violência autoprovocada*.*

## Método

### Tipo de estudo

Estudo metodológico de desenvolvimento e validação de um instrumento sobre as barreiras e facilitadores para a prevenção da violência autoprovocada, a partir dos referenciais teóricos de elaboração de escalas^(16)^ e TDF^(15)^. Os resultados foram descritos de acordo com *guidelines* PRISMA-COSMIN^(17)^.

### Cenário

O instrumento foi desenvolvido entre os meses de agosto e novembro de 2023. A validação de face e conteúdo ocorreu entre janeiro e fevereiro de 2024 e a validação de constructo entre junho e julho de 2024. Todo o processo de validação foi realizado por meio de coleta virtual, a partir da plataforma *Research Electronic Data Capture (REDCap).*

### Etapas do estudo

O processo de construção do instrumento seguiu procedimentos teóricos e experimentais, a fim de disponibilizar um instrumento passível para uso no cenário brasileiro^(16)^. Foram seguidas as etapas de definição dos objetivos e público-alvo do instrumento, definição das estruturas conceituais e operacionais baseadas no TDF^(15-16)^. O desenvolvimento dos itens do instrumento foi iniciado pelas autoras (AV, AS, LP), com revisão e discussões com autoras IM e KV. Na validação do instrumento, foram seguidas as etapas de validação de face, conteúdo e constructo, conforme descrito a seguir.

### População

A validação de face e conteúdo do instrumento foi realizada com 13 *experts*, enquanto a validação de constructo com 91 profissionais de saúde. Em ambas as etapas os participantes foram selecionados a partir da técnica de amostragem não-probabilística por conveniência.

### Critérios de seleção

A seleção dos *experts* foi realizada por meio da plataforma Lattes (sistema de informações sobre pesquisadores e instituições científicas, tecnológicas e de inovação do Brasil). Os critérios de inclusão foram apresentar formação na área da saúde, nível doutorado e experiência (docência, pesquisa ou assistencial) com a temática de violência autoprovocada. Os dados dos *experts* que contemplaram os critérios definidos foram salvos em documento editável, o qual foi utilizado para a realização dos convites na etapa de coleta de dados.

Para a seleção do público-alvo, foi definida a participação de profissionais de saúde com atuação no contexto brasileiro, de modo a permitir que o instrumento fosse válido para pessoas com diferentes níveis e experiências profissionais. Os critérios de inclusão foram ser profissional de saúde, brasileiro, com atuação profissional no país.

### Coleta de dados

A coleta de dados para as etapas de validação de face, conteúdo e constructo do estudo foram realizadas na plataforma REDCap. Ao serem convidados para a pesquisa, ambos (*experts* e público-alvo) tiveram acesso a um formulário na plataforma que continha o detalhamento da pesquisa, o Termo de Consentimento Livre e Esclarecido (TCLE), um questionário de caracterização sociodemográfica e o instrumento para identificação de barreiras e facilitadores para a prevenção da violência autoprovocada.

Na validação de face e conteúdo, foram convidados 80 *experts* por meio de *e-mails* com informações sobre a pesquisa. Destes, 30 demonstraram interesse em colaborar e 24 responderam os questionários. A amostra final foi composta após exclusão de questionários com respostas incompletas ou preenchimento inadequado dos itens de avaliação do instrumento (n = 13).

Para a seleção do público-alvo, profissionais de saúde foram convidados de forma ampla, por meio de postagens divulgadas nas redes sociais (*Instagram* e *Facebook* do grupo de pesquisa e pesquisadores responsáveis pelo estudo). Cento e vinte e um (n = 121) profissionais acessaram o questionário no *REDCap*. Destes, 98 finalizaram totalmente as respostas, sendo excluídos os questionários com 15% das respostas faltantes (n = 7). A amostra foi, então, composta por 91 profissionais.

A respeito dos *experts*, foram coletadas variáveis de caracterização sociodemográfica, como gênero, idade, raça/cor, localização geográfica (região e estado), nível de escolaridade, formação acadêmica, tempo de experiência profissional, área de atuação, experiência com relação à docência, assistência e pesquisa, com enfoque na violência autoprovocada e/ou saúde mental. Além disso, o instrumento de barreiras e facilitadores para a prevenção da violência autoprovocada continha as opções de resposta “totalmente adequado”, “adequado”, “neutro”, “inadequado” e “totalmente inadequado”. Ao final do instrumento, os *experts* tiveram espaço para sugestões de modificação dos itens do instrumento.

Já para o público-alvo, foram coletadas as mesmas variáveis de caracterização sociodemográfica supracitadas, bem como o instrumento com escala de resposta “concordo totalmente”, “concordo”, “neutro”, “discordo” e “discordo totalmente”. Além disso, foram coletadas variáveis relacionadas à facilidade de preenchimento do instrumento, compreensão dos itens de opções de resposta, além da relevância do instrumento para prática clínica e sugestões para modificação do mesmo quando pertinente.

### Tratamento e análise dos dados

Os dados obtidos foram tratados em planilha do *Microsoft Excel* 10 e analisados pelo programa R (*R core Team*, 2023) versão 4.3.1 com o uso do pacote *Psych* ^(18)^. A validade de face e conteúdo foi avaliada por meio de análises descritivas e pelo Índice de Validade de Conteúdo (IVC) com critério mínimo de aceitação ou reformulação igual, ou maior que 0,80^(19)^. A adequação foi calculada a partir do agrupamento das opções “totalmente adequado”, “adequado” e “neutro”. O grau de confiabilidade e concordância entre os avaliadores foi avaliado por meio do *First-order Agreement Coefficient* (AC1). De acordo com o teste, quanto mais próximo de 1,0 o resultado, maior é a concordância e confiabilidade entre *experts* ^(20)^.

A validade de construto foi avaliada considerando a dimensionalidade do instrumento, por meio da análise fatorial exploratória (AFE). A AFE foi utilizada para identificar a estrutura subjacente dos itens do instrumento e determinar o número de fatores que melhor representam os dados. A Análise Paralela de Horn objetivou definir a estrutura de correlações entre os itens e definir um conjunto de dimensões que representam essas variáveis. O Kaiser Meyer Olkin (KMO) foi conduzido para verificar se os dados eram adequados para serem submetidos a análise fatorial. No KMO, os valores obtidos podem variar de zero a um, sendo que valores próximos a zero indicam que a análise fatorial é inadequada^(21)^. Os valores < 0,5 são considerados inaceitáveis, valores entre 0,5 e 0,7 são considerados medíocres; valores entre 0,7 e 0,8 são considerados bons e valores > 0,8 são considerados ótimos e > 0,9 excelentes^(22)^.

O teste de esfericidade de Bartlett também foi utilizado para avaliar a adequação da análise fatorial por meio da avaliação da presença de correlação entre as variáveis, sendo significativa quando o valor entre os elementos da diagonal principal é igual a um, e ausentes quando os demais elementos da matriz ainda são próximo de zero^(23)^. O teste avalia também a significância geral de todas as correlações, sendo p < 0,05 indicativo de que a matriz é favorável a fatoração de dados^(24)^.

Após definição de fatores, foram excluídos da análise fatorial valores iguais ou inferiores a 0,40, por serem considerados insuficientes, ou seja, não carregarem o fator identificado. Por fim, o ω de McDonald definiu a fidedignidade da análise realizada.

### Aspectos éticos

O estudo foi aprovado pelo Comitê de Ética em Pesquisa da Escola de Enfermagem de Ribeirão Preto da Universidade de São Paulo (CEP/EERP-USP) sob parecer número 6.308.906.

## Resultados

O estudo foi desenvolvido em duas etapas principais, sendo estas a) desenvolvimento do instrumento e b) validação do instrumento, sendo a segunda etapa dividida entre validação de face/conteúdo e validação de constructo.

### Desenvolvimento do instrumento

Inicialmente foram desenvolvidas afirmações relacionadas à prevenção da violência autoprovocada foram desenvolvidas para cada domínio (n = 14) e constructos do TDF (n = 84), em seguida, as afirmações foram agrupadas e sintetizadas, resultando em 17 itens, além de duas questões discursivas para a flexibilização da resposta e adição de possíveis aspectos não abordados anteriormente. Uma escala Likert de cinco pontos (Concordo totalmente, Concordo, Neutro, Discordo, Discordo Totalmente) foi definida como escala de resposta para o instrumento^(25-26)^.

Os domínios relacionados ao TDF mantidos ao final da construção do instrumento “Barreiras e facilitadores para a prevenção da violência autoprovocada” foram: Conhecimento (n = 1), Capacidades (n = 1), Identidade/papel profissional (n = 1), Crenças sobre capacidades (n = 2), Crenças sobre consequências (n = 1), Reforço (n = 1), Metas (n = 1), Contexto ambiental e recursos (n = 3), Influências sociais (n = 3), Emoção (n = 2), Regulação comportamental (n = 1).

### Validação de face e conteúdo com *experts*

A presente etapa objetivou avaliar a adequação, apresentação e compreensão do instrumento^(16,27)^. A amostra final de participantes (n = 13) foi composta por profissionais autodeclarados do sexo feminino (76,8%), de raça branca (76,9%), da região sudeste (69,2%), com formação em enfermagem (92,3%) e níveis de doutorado ou pós-doutorado (69,3%). A maioria dos participantes tinham experiência profissional no campo da docência (84,6%) em pesquisa (100%) e na assistência (76,9%). Além disso, os profissionais relataram experiência na temática da violência autoprovocada (69,2%), e no campo da saúde mental na atenção primária à saúde (76,9%). 35,7% possuíam experiência prévia em estudos de construção e/ou validação de instrumentos.

Após análise pelos *experts*, apenas os itens 11, 12 e 15 não atingiram o IVC de 80% (IVC = 0,69; IVC = 0,77 e IVC = 0,77, respectivamente) (Tabela 1), e foram aprimorados de acordo com as sugestões recebidas.

No item 11, os *experts* solicitaram a adequação do termo “*as pessoas* que admiro” para “*os profissionais* que admiro”, para o melhor enfoque nas relações do ambiente de trabalho. No item 12, foi sugerida a substituição de “*desperta* ansiedade, medo…” para “*me desperta* ansiedade, medo…”. No item 15 os *experts* sugeriram maior especificidade e clareza e a questão foi modificada de “*sei quais serviços da rede*…” para “*sei quais serviços da rede* de atenção à saúde e outros dispositivos sociais…”.

Além disso, considerando as sugestões propostas no campo “Sugestões” do questionário apresentado aos *experts*, foram adicionados outros dois itens relacionados ao domínio “Contexto Ambiental e Recursos”.

**Tabela 1-:** Confiabilidade e concordância dos *experts* sobre o instrumento “Barreiras e facilitadores para a prevenção da violência autoprovocada” (n = 13). Ribeirão Preto, SP, Brasil, 2024

**Itens**	Concordância Adequação N (%)*							IVC**
	**TA** ^†^	**A** ^‡^	**N** ^§^	**I** ^ǁ^	**TI** ^¶^			
Item 1	12 (92,3)	1 (7,7)	-	-	-	1,00
Item 2	10 (76,9)	1 (7,7)	1 (7,7)	1 (7,7)	-	0,85
Item 3	11 (84,6)	1 (7,7)	1 (7,7)	-	-	0,92
Item 4	10 (76,9)	2 (15,4)	1 (7,7)	-	-	0,92
Item 5	10 (76,9)	2 (15,4)	-	1 (7,7)	-	0,92
Item 6	10 (76,9)	1 (7,7)	2 (15,4)	-	-	0,85
Item 7	9 (69,2)	2 (15,4)	1 (7,7)	-	1 (7,7)	0,85
Item 8 ^††^	8 (66,7)	2 (16,7)	1 (8,3)	1 (8,3)	-	0,83
Item 9	12 (92,3)	1 (7,7)	-	-	-	1,00
Item 10	11 (84,6)	-	-	2 (15,4)	-	0,85
Item 11	8 (61,5)	1 (7,7)	2 (15,4)	2 (15,4)	-	**0,69**
Item 12	9 (69,2)	1 (7,7)	1 (7,7)	1 (7,7)	1 (7,7)	**0,77**
Item 13	9 (69,2)	3 (23,1)	-	1 (7,7)	-	0,92
Item 14 ^††^	7 (58,3)	3 (25,0)	1 (8,3)	1 (8,3)	-	0,83
Item 15	9 (69,2)	1 (7,7)	1 (7,7)	1 (7,7)	1 (7,7)	**0,77**
Item 16	10 (76,9)	1 (7,7)	-	2 (15,4)	-	0,85
Item 17	11 (84,6)	-	-	2 (15,4)	-	0,85

*N = Número de participantes; ^†^TA = Totalmente adequado; ^‡^A = Adequado; ^§^N = Neutro; ^ǁ^I = Inadequado; ^¶^TI = Totalmente inadequado; **IVC = Índice de validade de conteúdo; ^††^Item com *missing*

### Validação de constructo com o público-alvo

A validação de constructo objetiva avaliar se os itens do instrumento realmente medem o conceito teórico que se propõe a medir. Dos 91 participantes, a maioria eram autodeclarados do sexo feminino (92,3%), da raça branca (74,4%), da região sudeste (85,6%), graduados em enfermagem (71,1%), com pós-graduação *Lato* ou *Stricto sensu* (48,4%), atuantes no setor público (61,5). Os participantes informaram não terem realizado formação prévia na temática da violência autoprovocada (65,9%), já terem atendido uma vítima de violência autoprovocada (86,7%) e negaram terem desenvolvido ou participado de uma ação de prevenção da violência autoprovocada (56%).

O teste de esfericidade de Bartlett foi significativo (p < 0,001) e o valor de KMO geral do instrumento foi 0,73, indicando boa adequação dos dados para a análise fatorial. Os itens 5 (crenças sobre consequências), 13 (influência sociais) e 14 (emoções) foram excluídos por possuírem KMO < 0,6 (Tabela 2).

**Tabela 2-:** Concordância de profissionais da saúde na validação de constructo do instrumento “Barreiras e facilitadores para a prevenção da violência autoprovocada” (n = 91). Ribeirão Preto, SP, Brasil, 2024

**Itens**	Concordância N (%)*							**KMO** **
	**CT** ^†^	**C** ^‡^	**N** ^§^	**D** ^ǁ^	**DT** ^¶^			
Item 1	7 (7,8)	35 (38,9)	23 (25,6)	13 (14,4)	12 (13,3)	0,72
Item 2	5 (5,6)	30 (33,3)	14 (15,6)	23 (25,6)	18 (20)	0,77
Item 3	11 (12,1)	23 (25,3)	18 (19,8)	25 (27,5)	14 (15,4)	0,82
Item 4	43 (47,8)	34 (37,8)	8 (8,9)	2 (2,2)	3 (3,3)	0,79
Item 5	70 (76,9)	20 (22)	1 (1,1)	-	-	**0,55**
Item 6	14 (15,4)	13 (14,3)	42 (46,2)	7 (7,7)	15 (16,5)	0,69
Item 7	4 (4,4)	5 (5,5)	15 (16,5)	24 (26,4)	43 (47,3)	0,73
Item 8	3 (3,3)	14 (15,4)	17 (18,7)	24 (26,4)	33 (36,3)	0,71
Item 9	1 (1,1)	20 (22,2)	19 (21,1)	27 (30)	23 (25,6)	0,72
Item 10	5 (5,5)	29 (31,9)	18 (19,8)	20 (22)	19 (20,9)	0,80
Item 11	18 (19,8)	46 (50,5)	21 (23,1)	4 (4,4)	2 (2,2)	0,68
Item 12	8 (8,8)	22 (24,2)	23 (25,3)	20 (22)	18 (19,8)	0,68
Item 13	38 (41,8)	44 (48,4)	-	8 (8,8)	1 (1,1)	**0,57**
Item 14	8 (8,9)	13 (14,4)	26 (28,9)	29 (32,2)	14 (15,6)	**0,54**
Item 15	21 (23,1)	42 (46,2)	14 (15,4)	13 (14,3)	1 (1,1)	0,79
Item 16	6 (6,6)	28 (30,8)	24 (26,4)	22 (24,2)	11 (12,1)	0,74
Item 17	21 (23,1)	30 (33)	15 (16,5)	18 (19,8)	7 (7,7)	0,87

*N = Número de participantes; ^†^CT = Concordo totalmente; ^‡^C = Concordo; ^§^N = Neutro; ^ǁ^D = Discordo; ^¶^DT = Discordo totalmente; **KMO = Teste de Kaiser-Meyer-Olkin

A análise paralela de Horn sugeriu a retenção de três fatores (conhecimentos e capacidades, contexto e apoio institucional), considerando estes os fatores pertinentes ao instrumento (Figura 1), sugerindo a exclusão dos itens 4 (identidade profissional), 6 (reforço), 11 (emoções), 12 (influências sociais) e confirmando a dimensionalidade esperada.

**Figura 1-:** Gráfico de análise paralela para definição do número de fatores pertencentes ao instrumento “Barreiras e facilitadores para a prevenção da violência autoprovocada”

A Tabela 3 mostra o resultado dos fatores (n = 3) e itens mantidos no instrumento final após teste de rotação oblíqua (*Oblimin*) e teste do método de extração.

**Tabela 3-:** Matriz de cargas fatoriais do instrumento “Barreiras e facilitadores para a prevenção da violência autoprovocada”. Ribeirão Preto, SP, Brasil, 2024

**Fator**		**Itens**	MR1*	**MR2**	**MR3**
1	Item 2		0,9161	0,0746	- 0,1050
	Item 1		0,8241	0,1107	- 0,0716
	Item 3		0,7992	- 0,0396	0,0293
	Item 16		0,6929	- 0,0974	0,1575
	Item 15		0,6122	- 0,1319	0,1180
	Item 17		0,5403	- 0,0297	0,2700
					
2	Item 9		0,0515	0,9111	0,0367
	Item 18		- 0,1218	0,6045	0,0289
	Item 10		0,0942	0,5640	0,1150
					
3	Item 7		0,0749	- 0,0308	0,7390
	Item 8		- 0,0744	0,2479	0,7110
*SS loadings* ^†^			3,3752	1,7282	1,3468
*Proportion Var* ^‡^			0,3068	0,1571	0,1224
*Cumulative Var* ^§^			0,3068	0,4639	**0,5864**

*Fator; ^†^ *SS loadings* = *Sum of squared loadings*; ^‡^ *Proportion Var* = *Proportion variance*; ^§^ *Cumulative Var* = *Cumulative variance*

A consistência interna do instrumento foi avaliada por meio do ω de McDonald e considerada boa (ω = 0,89). Os itens finais foram, então, renumerados e agrupados em três fatores que foram então nomeados para melhor representarem seus domínios (Figura 2).

**Figura 2-:** Fatores e respectivos itens componentes do instrumento “Barreiras e facilitadores para a prevenção da violência autoprovocada”. Ribeirão Preto, SP, Brasil, 2024

**Fator**	**Itens**
**1. Capacidade e conhecimento**	1. **Eu tenho preparo (técnico) para realizar** ações de prevenção da violência autoprovocada.
	2. **Eu sei como realizar** ações de prevenção da violência autoprovocada
	3. **Me sinto seguro(a) e capaz para desenvolver** ações de prevenção da violência autoprovocada.
	4. **Tenho segurança na conduta de cuidados** a uma vítima de violência autoprovocada no serviço onde trabalho.
	5. **Busco formas de aprimorar meu desempenho** para contribuir com a prevenção da violência autoprovocada.
	6. **Sei quais serviços da rede (de atenção à saúde e outros dispositivos sociais)** devo acionar para trabalhar a prevenção da violência autoprovocada.
**2. Contexto**	7. **Meu local de trabalho possui recursos físicos e materiais para** realizar ações de prevenção da violência autoprovocada.
	8. **Os serviços** **de saúde disponíveis na rede em que trabalho são suficientes** para atender as demandas ligadas à violência autoprovocada.
	9. **Meu local de trabalho** **possui recursos humanos para realização de ações** de prevenção da violência autoprovocada.
**3. Apoio institucional (reforço)**	10. **Em meu local de trabalho, existe alguma meta/indicador relacionados à** prevenção da violência autoprovocada.
	11. **Em meu local de trabalho existe um protocolo** para prevenção da violência autoprovocada.

## Discussão

O presente estudo desenvolveu e validou um instrumento para a identificação de barreiras e facilitadores para a prevenção da violência autoprovocada, baseado no TDF para uso no contexto brasileiro. A construção baseada no TDF se justifica pela escassez de instrumentos brasileiros com alto rigor teórico-metodológico, desenvolvidos para avaliação das barreiras e facilitadores para a prevenção da violência autoprovocada no contexto brasileiro.

O TDF propicia fundamentação cientificamente criteriosa para a identificação de fatores que podem atuar como barreiras ou facilitadores no comportamento de profissionais, refletindo em sua prática. Tal identificação contribui para o mapeamento de pontos estratégicos, que podem favorecer a implementação de práticas baseadas em evidências, ou outras mudanças desejáveis em serviços de saúde^(28-29)^.

Embora majoritariamente utilizado no desenvolvimento de instrumentos qualitativos, o TDF vem se mostrando cada vez mais presente na construção de questionários quantitativos^(30-32)^. Os itens desenvolvidos com escala resposta do tipo Likert, podem facilitar a coleta de dados quantitativos e passíveis de generalização, possibilitando comparações entre diferentes regiões do país. O instrumento apresentado tem como vantagem a combinação entre a quantidade reduzida de itens e a abrangência de constructos do TDF. Ainda, os qualitativos no instrumento possibilitam maior compreensão de fatores percebidos pelos participantes, promovendo a oportunidade para que os profissionais exponham especificidades de sua experiência e local de atuação. O levantamento dessas informações em diferentes contextos pode gerar *insights* relevantes para implementar intervenções que enfoquem fatores modificáveis e facilitem a implementação de estratégias de prevenção^(33-34)^.

Destaca-se que a literatura internacional aponta que as principais barreiras e facilitadores conhecidos, ligados à prevenção da violência autoprovocada, estão relacionadas à formação de profissionais, crenças e atitudes, cultura institucional, recursos financeiros e humanos, apoio institucional, ferramentas de avaliação, políticas públicas e o acesso à assistência de qualidade^(10-14)^. O instrumento desenvolvido neste estudo permite a avaliação desses e de outros fatores que podem contribuir ou prejudicar a prevenção do suicídio em diferentes contextos.

Uma vantagem adicional do instrumento é que as afirmações que compõem cada item podem ser parcialmente modificadas. Todos os itens são compostos por duas partes, sendo uma delas em negrito, a qual especifica a característica do domínio do TDF^(15)^ outra parte que menciona “ações de prevenção da violência autoprovocada”, sendo que essa parte pode ser adaptada para ser direcionada a ações específicas de prevenção, tais como planos de gestão de crises, psicoeducação para pais, atividades de prevenção em escolas, entre outros. Desse modo, o instrumento pode avaliar as ações preventivas (de modo geral) ou pode ter todos os itens parcialmente adaptados, para enfocar apenas uma ação, como, por exemplo, o plano de gestão de crises. O item 1 “*Eu sei como realizar* ações de prevenção da violência autoprovocada” poderia ser adaptado para “*Eu sei como realizar* planos de gestão de crises” e os demais itens poderiam ser modificados de forma semelhante.

As limitações do estudo estão ligadas ao fato das avaliações do instrumento serem realizadas por profissionais predominantemente da região Sudeste do Brasil. O Brasil possui extenso território geográfico, composto por diversidade e singularidades, sendo relevante que o instrumento também possa identificar as barreiras e facilitadores para a prevenção da violência autoprovocada em todas as regiões do país.

Este estudo traz contribuições relevantes para a ciência da implementação, políticas públicas e melhoria dos cuidados em saúde mental, pois disponibiliza um instrumento gratuitamente, validado para o contexto brasileiro. O instrumento é de domínio público e pode ser utilizado por outros pesquisadores em todo o país, sem a necessidade de autorização prévia das autoras (desde que o estudo apropriadamente citado). O acesso aberto ao instrumento pode facilitar que pesquisadores das diferentes regiões do Brasil utilizem o instrumento em seus contextos.

## Conclusão

Com base nos resultados obtidos, conclui-se que o instrumento apresenta boa adequação e consistência interna, indicando que o instrumento é confiável para medir os constructos propostos. Após avaliação por *experts* na temática e público-alvo do instrumento, a versão final foi composta por 11 questões para resposta em escala do tipo Likert e duas questões discursivas. O presente estudo resultou então um instrumento de autopreenchimento, fácil de uso e passível de adaptações para compreender as barreiras e facilitadores para a violência autoprovocada em contextos no Brasil. Esses resultados indicam a relevância e a robustez do instrumento para futuras pesquisas, estratégias de prevenção e políticas de saúde.

# Translation: Barreras y facilitadores para la prevención de la violencia autoinfligida: desarrollo y validación de un instrumento para profesionales de la salud*

**DOI:** 10.1590/1518-8345.7754.4637

**Authors:** Amanda Sarah Vanzela, Aline Conceição Silva, Laysa Fernanda Silva Pedrollo, Isabela dos Santos Martin, Kelly Graziani Giacchero Vedana

**Affiliations:** 1 Universidade de São Paulo, Escola de Enfermagem de Ribeirão Preto, Centro Colaborador de la OPS/OMS para el Desarrollo de la Investigación en Enfermería, Ribeirão Preto, SP, Brasil; 2 Becaria de la Coordenação de Aperfeiçoamento de Pessoal de Nível Superior (CAPES), Brasil; 3 Universidade de São Paulo, Escola de Enfermagem, São Paulo, SP, Brasil

**Keywords:** Prevención del Suicidio, Conducta Autodestructiva, Práctica Clínica Basada en la Evidencia, Estudio de Validación, Personal de Salud, Atención a la Salud

## Abstract

desarrollar y validar un instrumento basado en el *Theoretical Domains Framework* para la identificación de barreras y facilitadores percibidos por profesionales de la salud en la prevención de la violencia autoinfligida.

estudio metodológico basado en el marco teórico de Pasquali y en el *Theoretical Domains Framework*. Las validaciones de apariencia y contenido se realizaron mediante evaluación de expertos (n = 13), y la validez de constructo se verificó con profesionales de la salud (n = 91). Los participantes fueron seleccionados por muestreo no probabilístico por conveniencia. Los datos fueron recolectados mediante formulario virtual y analizados a partir del Índice de Validez de Contenido (IVC), el coeficiente de concordancia *First-Order Agreement* (AC1) y el análisis factorial exploratorio de Horn.

la concordancia entre evaluadores fue considerada moderada (AC1 = 0,52; DE = 0,04; IC 95% = 0,42 - 0,61; p ≤ 0,005). Tras el análisis con el público objetivo, se eliminaron tres ítems (KMO = < 0,6). El análisis factorial identificó tres factores y 11 ítems, y la consistencia interna del instrumento fue considerada alta (ω = 0,89). La versión final fue estructurada con 11 ítems en escala tipo Likert.

el instrumento es pionero y accesible para el contexto brasileño, con potencial para la identificación de barreras y facilitadores en la prevención de la violencia autoinfligida.

## Destacados

**(1)** El instrumento identifica barreras y facilitadores en la prevención de la violencia autoinfligida. **(2)** La consistencia interna del instrumento fue considerada alta. **(3)** El análisis identificó factores: conocimientos y capacidad, contexto y apoyo institucional. **(4)** Se desarrolló un instrumento de fácil utilización y adaptable a diversos contextos de Brasil. **(5)** El instrumento contribuye a los estudios de prevención de la violencia autoinfligida.

## Introducción

La violencia autoinfligida es compleja, multifactorial y representa un tema prioritario en las agendas de salud pública a nivel mundial^(1)^. A pesar de las diversas estrategias de prevención existentes, aún persisten numerosos desafíos en el desarrollo e implementación efectiva de intervenciones preventivas basadas en evidencias relevantes para realidades específicas de salud^(1-2)^.

La violencia autoinfligida se define como un tipo de violencia que una persona ejerce contra sí misma, e incluye el comportamiento suicida (intentos y suicidio), además de las autolesiones no suicidas (ALNS)^(2)^. Cada año, miles de personas mueren por suicidio en todo el mundo^(1)^; en Brasil, en 2021, se registraron más de 15 mil muertes^(3)^. Se estima que los intentos de suicidio y las ALNS son aún más frecuentes y numerosas, aunque subnotificadas.

Por tratarse de un problema multifacético, la prevención de la violencia autoinfligida debe ser concebida y organizada a partir de un enfoque multinivel y multiprofesional^(1,4)^. La Organización Mundial de la Salud (OMS) propone que la restricción de métodos letales, la educación y capacitación de profesionales de la salud, los programas de intervención en crisis, el manejo de trastornos mentales, la formación de profesionales de los medios de comunicación y la posvención son estrategias eficaces y necesarias para la prevención de la violencia autoinfligida^(5-6)^.

Sin embargo, la existencia de estrategias efectivas de prevención no garantiza su debida implementación y aplicación en la realidad de los profesionales de salud. Existe una gran brecha entre lo que se produce desde la ciencia y lo que realmente se aplica en la práctica^(7-8)^. En este contexto, es necesario comprender cómo las estrategias basadas en evidencias son y pueden ser efectivamente implementadas en la práctica^(9)^. Por lo tanto, en primer lugar, es importante identificar el contexto en el que se desarrollará una práctica, así como los factores que influyen o no en su implementación y resultados^(7)^.

Investigaciones en diferentes países buscan identificar las barreras y facilitadores para la implementación de estrategias de prevención de la violencia autoinfligida^(10-11)^. Las principales barreras vinculadas a la prevención de la violencia por parte de profesionales de la Atención Primaria de Salud (APS) son la falta de formación y educación de los profesionales, creencias y actitudes^(12)^, estigma y juicios^(11,13)^, aspectos relacionados con la cultura institucional^(12)^, así como limitaciones vinculadas a los recursos financieros y humanos^(11,13)^. Los principales facilitadores identificados son la formación de los profesionales de salud, el apoyo institucional^(10-11)^, la disponibilidad de herramientas de evaluación adecuadas y contextualizadas^(10-11)^, políticas públicas que valoren los derechos de las víctimas de violencia y el acceso a una atención de calidad^(14)^.

Es necesario que estos estudios sobre implementación de prácticas se desarrollen y se fundamenten en el contexto específico en el cual se pretende implementar la estrategia^(9)^. En Brasil, aún no existen instrumentos que propongan identificar barreras y facilitadores para la prevención de la violencia autoinfligida desde la perspectiva de los profesionales de la salud. Los estudios de esta naturaleza aún carecen de fundamentos en marcos teóricos que sustenten su comprensión.

El *Theoretical Domains Framework* (TDF) es una estructura teórica integradora construida por psicólogos e investigadores expertos en cambio de comportamiento e implementación de prácticas basadas en evidencias. La estructura se fundamenta en 33 teorías de la psicología y tiene como objetivo identificar los factores que influyen en los comportamientos de los profesionales de la salud para implementar o no (barreras y facilitadores) prácticas basadas en evidencias^(15)^.

La utilización de este tipo de herramienta puede contribuir a la identificación de barreras y facilitadores, favoreciendo la implementación de estrategias de prevención de la violencia autoinfligida basadas en evidencias, promoviendo la mejora de las prácticas preventivas y de la calidad del trabajo en salud, así como de la atención a víctimas y sobrevivientes. Estos hallazgos presentan potencial para fomentar discusiones sobre esta temática, con enfoque en cambios relacionados con la formulación de políticas públicas de prevención de la violencia autoinfligida en Brasil. Así, el presente estudio tuvo como objetivo desarrollar y validar un instrumento basado en el TDF para la identificación de barreras y facilitadores percibidos por profesionales de la salud en la prevención de la violencia autoinfligida.

## Método

### Tipo de estudio

Estudio metodológico de desarrollo y validación de un instrumento sobre las barreras y facilitadores para la prevención de la violencia autoinfligida, basado en los marcos teóricos de elaboración de escalas^(16)^ y en el TDF^(15)^. Los resultados fueron descritos de acuerdo con las directrices PRISMA-COSMIN^(17)^.

### Escenario

El instrumento fue desarrollado entre los meses de agosto y noviembre de 2023. La validación de apariencia y contenido se llevó a cabo entre enero y febrero de 2024, y la validación de constructo entre junio y julio de 2024. Todo el proceso de validación se realizó mediante recolección virtual, mediante la plataforma *Research Electronic Data Capture* (*REDCap*).

### Etapas del estudio

El proceso de construcción del instrumento siguió procedimientos teóricos y experimentales con el fin de ofrecer un instrumento aplicable en el contexto brasileño^(16)^. Se siguieron las etapas de definición de los objetivos y del público objetivo del instrumento, así como la definición de estructuras conceptuales y operacionales basadas en el TDF^(15-16)^. El desarrollo de los ítems del instrumento fue iniciado por las autoras (AV, AS, LP), con revisión y discusión junto a las autoras IM y KV. En la validación del instrumento, se siguieron las etapas de validación de apariencia, contenido y constructo, conforme se describe a continuación.

### Población

La validación de apariencia y contenido del instrumento se realizó con 13 expertos, mientras que la validación de constructo se llevó a cabo con 91 profesionales de la salud. En ambas las etapas, los participantes fueron seleccionados mediante técnica de muestreo no probabilístico por conveniencia.

### Criterios de selección

La selección de los expertos se realizó mediante la *Plataforma Lattes* (sistema de información sobre investigadores e instituciones científicas, tecnológicas e innovadoras de Brasil). Los criterios de inclusión fueron poseer formación en el área de la salud, nivel de doctorado y experiencia (docencia, investigación o asistencia) en la temática de violencia autoinfligida. Los datos de los expertos que cumplieron con los criterios definidos fueron guardados en un documento editable, el cual fue utilizado para la realización de las invitaciones en la etapa de recolección de datos.

Para la selección del público objetivo, se definió la participación de profesionales de salud que actuaran en el contexto brasileño, con el fin de permitir que el instrumento fuera válido para personas con diferentes niveles y experiencias profesionales. Los criterios de inclusión fueron ser profesional de salud, brasileño y con actuación profesional en el país.

### Recolección de datos

La recolección de datos para las etapas de validación de apariencia, contenido y constructo del estudio se realizó en la plataforma *REDCap*. Al ser invitados a participar en la investigación, tanto expertos como público objetivo tuvieron acceso a un formulario en la plataforma que contenía la descripción detallada del estudio, el Consentimiento Informado (CI), un cuestionario de caracterización sociodemográfica y el instrumento para la identificación de barreras y facilitadores para la prevención de la violencia autoinfligida.

En la validación de apariencia y contenido, se invitó a 80 expertos mediante correos electrónicos con información sobre el estudio. De estos, 30 manifestaron interés en colaborar y 24 respondieron los cuestionarios. La muestra final fue compuesta tras la exclusión de cuestionarios con respuestas incompletas o con un llenado inadecuado de los ítems de evaluación del instrumento (n = 13).

Para la selección del público objetivo, se invitó ampliamente a profesionales de salud mediante publicaciones en redes sociales (*Instagram* y *Facebook* del grupo de investigación y de los investigadores responsables por el estudio). Un total de 121 profesionales accedieron al cuestionario en *REDCap*. De estos, 98 completaron todas las respuestas, siendo excluidos los cuestionarios con 15% de respuestas faltantes (n = 7). La muestra quedó compuesta por 91 profesionales.

Respecto a los expertos, se recolectaron variables de caracterización sociodemográfica como género, edad, raza/color, ubicación geográfica (región y estado), nivel educativo, formación académica, tiempo de experiencia profesional, área de actuación, experiencia en docencia, asistencia e investigación, con enfoque en violencia autoinfligida y/o salud mental. Además, el instrumento de barreras y facilitadores para la prevención de la violencia autoinfligida contenía las opciones de respuesta “totalmente adecuado”, “adecuado”, “neutro”, “inadecuado” y “totalmente inadecuado”. Al final del instrumento, los expertos disponían de un espacio para sugerencias de modificación de los ítems del instrumento.

Para el público objetivo, se recolectaron las mismas variables de caracterización sociodemográfica mencionadas anteriormente, así como el instrumento con escala de respuesta “totalmente de acuerdo”, “de acuerdo”, “neutro”, “en desacuerdo” y “totalmente en desacuerdo”. Además, se recolectaron variables relacionadas con la facilidad de llenado del instrumento, comprensión de los ítems y opciones de respuesta, así como la relevancia del instrumento para la práctica clínica y sugerencias de modificación del mismo cuando fuera pertinente.

### Tratamiento y análisis de los datos

Los datos obtenidos fueron organizados en una hoja de cálculo de *Microsoft Excel* 10 y analizados con el programa R (*R Core Team*, 2023), versión 4.3.1, utilizando el paquete *Psych* ^(18)^. La validez de apariencia y contenido fue evaluada mediante análisis descriptivos y el Índice de Validez de Contenido (IVC), con un criterio mínimo de aceptación o reformulación igual o superior a 0,80^(19)^. La adecuación se calculó a partir del agrupamiento de las opciones “totalmente adecuado”, “adecuado” y “neutro”. El grado de confiabilidad y concordancia entre los evaluadores fue evaluado mediante el coeficiente *First-order Agreement* (AC1). Según esta prueba, cuanto más cercano a 1,0 sea el resultado, mayor es la concordancia y confiabilidad entre los expertos^(20)^.

La validez de constructo fue evaluada considerando la dimensionalidad del instrumento, mediante análisis factorial exploratorio (AFE). La AFE se utilizó para identificar la estructura subyacente de los ítems del instrumento y determinar el número de factores que mejor representan los datos. El Análisis Paralelo de Horn tuvo como objetivo definir la estructura de correlaciones entre los ítems y establecer un conjunto de dimensiones que representen esas variables. Se aplicó el índice Kaiser-Meyer-Olkin (KMO) para verificar si los datos eran adecuados para someterse al análisis factorial. En el KMO, los valores obtenidos pueden variar entre cero y uno, siendo que valores cercanos a cero indican que el análisis factorial es inadecuado^(21)^. Valores < 0,5 se consideran inaceptables; valores entre 0,5 y 0,7, mediocres; valores entre 0,7 y 0,8, buenos; valores > 0,8, óptimos; y valores > 0,9, excelentes^(22)^.

La prueba de esfericidad de Bartlett también fue utilizada para evaluar la adecuación del análisis factorial mediante la verificación de la existencia de correlación entre las variables, siendo significativa cuando los valores de los elementos en la diagonal principal son iguales a uno y están ausentes cuando los demás elementos de la matriz se aproximan a cero^(23)^. La prueba también evalúa la significancia general de todas las correlaciones, considerándose p < 0,05 como indicativo de que la matriz es favorable para la factorización de los datos^(24)^.

Tras la definición de factores, se excluyeron del análisis factorial los valores iguales o inferiores a 0,40 por ser considerados insuficientes, es decir, por no cargar el factor identificado. Por último, el coeficiente ω de McDonald determinó la fidelidad del análisis realizado.

### Aspectos éticos

El estudio fue aprobado por el Comité de Ética en Investigación de la Escuela de Enfermería de Ribeirão Preto de la Universidad de São Paulo (CEP/EERP-USP), con parecer número 6.308.906.

## Resultados

El estudio se desarrolló en dos etapas principales: a) desarrollo del instrumento y b) validación del instrumento, siendo esta segunda etapa dividida en validación de apariencia/contenido y validación de constructo.

### Desarrollo del instrumento

Inicialmente se desarrollaron afirmaciones relacionadas con la prevención de la violencia autoinfligida para cada dominio (n = 14) y constructos del TDF (n = 84). Posteriormente, las afirmaciones fueron agrupadas y sintetizadas, resultando en 17 ítems, además de dos preguntas abiertas para permitir la flexibilización de la respuesta y la inclusión de posibles aspectos no abordados anteriormente. Se definió una escala Likert de cinco puntos (Totalmente de acuerdo, De acuerdo, Neutro, En desacuerdo, Totalmente en desacuerdo) como escala de respuesta del instrumento^(25-26)^.

Los dominios relacionados con el TDF mantenidos al final de la construcción del instrumento “Barreras y facilitadores para la prevención de la violencia autoinfligida” fueron: Conocimiento (n = 1), Capacidades (n = 1), Identidad/rol profesional (n = 1), Creencias sobre capacidades (n = 2), Creencias sobre consecuencias (n = 1), Reforzamiento (n = 1), Metas (n = 1), Contexto ambiental y recursos (n = 3), Influencias sociales (n = 3), Emoción (n = 2), Regulación del comportamiento (n = 1).

### Validación de apariencia y contenido con expertos

La presente etapa tuvo como objetivo evaluar la adecuación, presentación y comprensión del instrumento^(16,27)^. La muestra final de participantes (n = 13) estuvo compuesta por profesionales autodeclaradas de sexo femenino (76,8%), de raza blanca (76,9%), de la región sudeste (69,2%), con formación en enfermería (92,3%) y con niveles de doctorado o posdoctorado (69,3%). La mayoría de las participantes tenía experiencia profesional en el ámbito de la docencia (84,6%), en investigación (100%) y en la asistencia (76,9%). Además, los profesionales informaron tener experiencia en la temática de la violencia autoinfligida (69,2%) y en el campo de la salud mental en la atención primaria de salud (76,9%). El 35,7% tenía experiencia previa en estudios de construcción y/o validación de instrumentos.

Tras el análisis por parte de los expertos, solo los ítems 11, 12 y 15 no alcanzaron el IVC del 80% (IVC = 0,69; IVC = 0,77 e IVC = 0,77, respectivamente) (Tabla 1), y fueron mejorados según las sugerencias recibidas.

En el ítem 11, los expertos solicitaron la adecuación del término “*las personas* que admiro” por “*los profesionales* que admiro”, para enfocar mejor las relaciones en el entorno laboral. En el ítem 12, se sugirió sustituir “*despierta* ansiedad, miedo...” por “*me despierta* ansiedad, miedo...”. En el ítem 15, los expertos sugirieron mayor especificidad y claridad, por lo que la pregunta fue modificada de “*sé cuáles son los servicios de la red*...” a “i de atención a la salud y otros dispositivos sociales...”.

Además, considerando las sugerencias propuestas en el campo “Sugerencias” del cuestionario presentado a los expertos, se añadieron otros dos ítems relacionados con el dominio “Contexto Ambiental y Recursos”.

**Tabla 1-:** Confiabilidad y concordancia de los expertos sobre el instrumento “Barreras y facilitadores para la prevención de la violencia autoinfligida” (n = 13). Ribeirão Preto, SP, Brasil, 2024

Ítems	Concordancia Adecuación N (%)*					IVC**
	**TA** ^†^	**A** ^‡^	**N** ^§^	**I** ^ǁ^	**TI** ^¶^	
Ítem 1	12 (92,3)	1 (7,7)	-	-	-	1,00
Ítem 2	10 (76,9)	1 (7,7)	1 (7,7)	1 (7,7)	-	0,85
Ítem 3	11 (84,6)	1 (7,7)	1 (7,7)	-	-	0,92
Ítem 4	10 (76,9)	2 (15,4)	1 (7,7)	-	-	0,92
Ítem 5	10 (76,9)	2 (15,4)	-	1 (7,7)	-	0,92
Ítem 6	10 (76,9)	1 (7,7)	2 (15,4)	-	-	0,85
Ítem 7	9 (69,2)	2 (15,4)	1 (7,7)	-	1 (7,7)	0,85
Ítem 8 ^††^	8 (66,7)	2 (16,7)	1 (8,3)	1 (8,3)	-	0,83
Ítem 9	12 (92,3)	1 (7,7)	-	-	-	1,00
Ítem 10	11 (84,6)	-	-	2 (15,4)	-	0,85
Ítem 11	8 (61,5)	1 (7,7)	2 (15,4)	2 (15,4)	-	**0,69**
Ítem 12	9 (69,2)	1 (7,7)	1 (7,7)	1 (7,7)	1 (7,7)	**0,77**
Ítem 13	9 (69,2)	3 (23,1)	-	1 (7,7)	-	0,92
Ítem 14 ^††^	7 (58,3)	3 (25,0)	1 (8,3)	1 (8,3)	-	0,83
Ítem 15	9 (69,2)	1 (7,7)	1 (7,7)	1 (7,7)	1 (7,7)	**0,77**
Ítem 16	10 (76,9)	1 (7,7)	-	2 (15,4)	-	0,85
Ítem 17	11 (84,6)	-	-	2 (15,4)	-	0,85

*N = Número de participantes; ^†^TA = Totalmente adecuado; ^‡^A = Adecuado; ^§^N = Neutro; ^ǁ^I = Inadecuado; ^¶^TI = Totalmente inadecuado; **IVC = Índice de validez de contenido; ^††^Ítem con *missing*

### Validación de constructo con el público objetivo

La validación de constructo tiene como objetivo evaluar si los ítems del instrumento realmente miden el concepto teórico que se propone medir. De los 91 participantes, la mayoría se autodeclararon de sexo femenino (92,3%), de raza blanca (74,4%), de la región sudeste (85,6%), graduados en enfermería (71,1%), con posgrado *Lato* o *Stricto sensu* (48,4%) y con actuación en el sector público (61,5%). Los participantes informaron no haber realizado formación previa en la temática de la violencia autoinfligida (65,9%), haber atendido alguna vez a una víctima de violencia autoinfligida (86,7%) y negaron haber desarrollado o participado en una acción de prevención de la violencia autoinfligida (56%).

La prueba de esfericidad de Bartlett fue significativa (p < 0,001) y el valor KMO general del instrumento fue de 0,73, lo que indica una buena adecuación de los datos para el análisis factorial. Los ítems 5 (creencias sobre consecuencias), 13 (influencias sociales) y 14 (emociones) fueron excluidos por presentar KMO < 0,6 (Tabla 2).

**Tabla 2-:** Concordancia de profesionales de la salud en la validación de constructo del instrumento “Barreras y facilitadores para la prevención de la violencia autoinfligida” (n = 91). Ribeirão Preto, SP, Brasil, 2024

Ítems	Concordancia N (%)*					**KMO** **
	**CT** ^†^	**C** ^‡^	**N** ^§^	**D** ^ǁ^	**DT** ^¶^	
Ítem 1	7 (7,8)	35 (38,9)	23 (25,6)	13 (14,4)	12 (13,3)	0,72
Ítem 2	5 (5,6)	30 (33,3)	14 (15,6)	23 (25,6)	18 (20)	0,77
Ítem 3	11 (12,1)	23 (25,3)	18 (19,8)	25 (27,5)	14 (15,4)	0,82
Ítem 4	43 (47,8)	34 (37,8)	8 (8,9)	2 (2,2)	3 (3,3)	0,79
Ítem 5	70 (76,9)	20 (22)	1 (1,1)	-	-	**0,55**
Ítem 6	14 (15,4)	13 (14,3)	42 (46,2)	7 (7,7)	15 (16,5)	0,69
Ítem 7	4 (4,4)	5 (5,5)	15 (16,5)	24 (26,4)	43 (47,3)	0,73
Ítem 8	3 (3,3)	14 (15,4)	17 (18,7)	24 (26,4)	33 (36,3)	0,71
Ítem 9	1 (1,1)	20 (22,2)	19 (21,1)	27 (30)	23 (25,6)	0,72
Ítem 10	5 (5,5)	29 (31,9)	18 (19,8)	20 (22)	19 (20,9)	0,80
Ítem 11	18 (19,8)	46 (50,5)	21 (23,1)	4 (4,4)	2 (2,2)	0,68
Ítem 12	8 (8,8)	22 (24,2)	23 (25,3)	20 (22)	18 (19,8)	0,68
Ítem 13	38 (41,8)	44 (48,4)	-	8 (8,8)	1 (1,1)	**0,57**
Ítem 14	8 (8,9)	13 (14,4)	26 (28,9)	29 (32,2)	14 (15,6)	**0,54**
Ítem 15	21 (23,1)	42 (46,2)	14 (15,4)	13 (14,3)	1 (1,1)	0,79
Ítem 16	6 (6,6)	28 (30,8)	24 (26,4)	22 (24,2)	11 (12,1)	0,74
Ítem 17	21 (23,1)	30 (33)	15 (16,5)	18 (19,8)	7 (7,7)	0,87

*N = Número de participantes; ^†^CT = Totalmente de acuerdo; ^‡^C = De acuerdo; ^§^N = Neutro; ^ǁ^D = En desacuerdo; ^¶^DT = Totalmente en desacuerdo; **KMO = Prueba de Kaiser-Meyer-Olkin

El análisis paralelo de Horn sugirió la retención de tres factores (conocimientos y capacidades, contexto y apoyo institucional), considerándose estos como los factores pertinentes del instrumento (Figura 1), lo que llevó a la exclusión de los ítems 4 (identidad profesional), 6 (reforzamiento), 11 (emociones), 12 (influencias sociales) y confirmó la dimensionalidad esperada.

La Tabla 3 presenta el resultado de los factores (n = 3) y los ítems mantenidos en el instrumento final tras la prueba de rotación oblicua (*Oblimin*) y el test del método de extracción.

**Figura 1-:** Cuadro de análisis paralelo para la definición del número de factores pertenecientes al instrumento “Barreras y facilitadores para la prevención de la violencia autoinfligida”

**Tabla 3-:** Matriz de cargas factoriales del instrumento “Barreras y facilitadores para la prevención de la violencia autoinfligida”. Ribeirão Preto, SP, Brasil, 2024

**Factor**	Ítems	MR1*	**MR2**	**MR3**
1	Ítem 2	0,9161	0,0746	0,1050
	Ítem 1	0,8241	0,1107	0,0716
	Ítem 3	0,7992	0,0396	0,0293
	Ítem 16	0,6929	0,0974	0,1575
	Ítem 15	0,6122	0,1319	0,1180
	Ítem 17	0,5403	0,0297	0,2700
			
2	Ítem 9	0,0515	0,9111	0,0367
	Ítem 18	0,1218	0,6045	0,0289
	Ítem 10	0,0942	0,5640	0,1150
			
3	Ítem 7	0,0749	-0,0308	0,7390
	Ítem 8	0,0744	0,2479	0,7110
*SS loadings* ^†^		3,3752	1,7282	1,3468
*Proportion Var* ^‡^		0,3068	0,1571	0,1224
*Cumulative Var* ^§^		0,3068	0,4639	**0,5864**

*Factor; ^†^ *SS loadings* = *Sum of squared loadings*; ^‡^ *Proportion Var* = *Proportion variance*; ^§^ *Cumulative Var* = *Cumulative variance*

La consistencia interna del instrumento fue evaluada mediante el coeficiente ω de McDonald y considerada buena (ω = 0,89). Los ítems finales fueron entonces renumerados y agrupados en tres factores, los cuales fueron nombrados para representar mejor sus respectivos dominios (Figura 2).

**Figura 2-:** Factores y respectivos ítems componentes del instrumento “Barreras y facilitadores para la prevención de la violencia autoinfligida”. Ribeirão Preto, SP, Brasil, 2024

**Factor**	Ítems
**1. Capacidad y conocimiento**	1. **Tengo preparación (técnica) para realizar** acciones de prevención de la violencia autoinfligida.
	2. **Sé cómo llevar a cabo** acciones de prevención de la violencia autoinfligida.
	3. **Me siento seguro(a) y capaz de desarrollar** acciones de prevención de la violencia autoinfligida.
	4. **Tengo seguridad en la conducta de atención** a una víctima de violencia autoinfligida en el servicio donde trabajo.
	5. **Busco formas de mejorar mi desempeño** para contribuir a la prevención de la violencia autoinfligida.
	6. **Sé cuáles servicios de la red (de atención a la salud y otros dispositivos sociales)** debo activar para trabajar la prevención de la violencia autoinfligida.
**2. Contexto**	7. **Mi lugar de trabajo cuenta con recursos físicos y materiales para** realizar acciones de prevención de la violencia autoinfligida.
	8. **Los servicios de salud disponibles en la red en la que trabajo son suficientes** para atender las demandas relacionadas con la violencia autoinfligida.
	9. **Mi lugar de trabajo cuenta con recursos humanos para la realización de acciones** de prevención de la violencia autoinfligida.
**3. Apoyo institucional (refuerzo)**	10. **En mi lugar de trabajo, existe alguna meta/indicador relacionado** con la prevención de la violencia autoinfligida.
	11. **En mi lugar de trabajo existe un protocolo** para la prevención de la violencia autoinfligida.

## Discusión

El presente estudio desarrolló y validó un instrumento para la identificación de barreras y facilitadores para la prevención de la violencia autoinfligida, basado en el TDF, para su uso en el contexto brasileño. La construcción basada en el TDF se justifica por la escasez de instrumentos brasileños con alto rigor teórico-metodológico desarrollados para evaluar las barreras y facilitadores en la prevención de la violencia autoinfligida en dicho contexto.

El TDF proporciona una fundamentación científicamente rigurosa para la identificación de factores que pueden actuar como barreras o facilitadores del comportamiento profesional, reflejándose en su práctica. Tal identificación contribuye al mapeo de puntos estratégicos que pueden favorecer la implementación de prácticas basadas en evidencia u otros cambios deseables en los servicios de salud^(28-29)^.

Aunque ha sido utilizado mayoritariamente en el desarrollo de instrumentos cualitativos, el TDF ha ganado espacio en la construcción de cuestionarios cuantitativos^(30-32)^. Los ítems desarrollados con escala de respuesta tipo Likert pueden facilitar la recolección de datos cuantitativos susceptibles de generalización, permitiendo comparaciones entre diferentes regiones del país. El instrumento presentado tiene como ventaja la combinación entre la cantidad reducida de ítems y la amplitud de constructos del TDF. Además, los elementos cualitativos presentes en el instrumento permiten una mayor comprensión de los factores percibidos por los participantes, promoviendo la posibilidad de que los profesionales expresen especificidades de su experiencia y del lugar donde actúan. La recopilación de esta información en distintos contextos puede generar hallazgos relevantes para implementar intervenciones enfocadas en factores modificables y facilitar la implementación de estrategias de prevención^(33-34)^.

Cabe destacar que la literatura internacional señala que las principales barreras y facilitadores conocidos en relación con la prevención de la violencia autoinfligida están relacionadas con la formación de profesionales, creencias y actitudes, cultura institucional, recursos financieros y humanos, apoyo institucional, herramientas de evaluación, políticas públicas y acceso a una atención de calidad^(10-14)^. El instrumento desarrollado en este estudio permite evaluar estos y otros factores que pueden contribuir o dificultar la prevención del suicidio en distintos contextos.

Una ventaja adicional del instrumento es que las afirmaciones que componen cada ítem pueden ser parcialmente modificadas. Todos los ítems constan de dos partes, siendo una de ellas en negrita, que especifica la característica del dominio del TDF^(15)^, y otra parte que menciona “acciones de prevención de la violencia autoinfligida”, la cual puede ser adaptada para referirse a acciones preventivas específicas, como planes de gestión de crisis, psicoeducación para padres, actividades de prevención en escuelas, entre otros. De este modo, el instrumento puede evaluar acciones preventivas de manera general o puede ser adaptado parcialmente para centrarse únicamente en una acción específica, como por ejemplo, el plan de gestión de crisis. El ítem 1 “*Sé cómo llevar a cabo* acciones de prevención de la violencia autoinfligida” podría ser adaptado a “*Sé cómo llevar a cabo* planes de gestión de crisis”, y los demás ítems podrían ser modificados de forma semejante.

Las limitaciones del estudio están relacionadas con el hecho de que las evaluaciones del instrumento fueron realizadas principalmente por profesionales de la región sudeste de Brasil. Brasil posee un extenso territorio geográfico, caracterizado por su diversidad y singularidades, por lo que resulta relevante que el instrumento también pueda identificar las barreras y facilitadores para la prevención de la violencia autoinfligida en todas las regiones del país.

Este estudio aporta contribuciones relevantes a la ciencia de la implementación, a las políticas públicas y a la mejora de los cuidados en salud mental, al poner a disposición un instrumento validado para el contexto brasileño de forma gratuita. El instrumento es de dominio público y puede ser utilizado por otros investigadores en todo el país sin necesidad de autorización previa de las autoras (siempre que el estudio sea citado adecuadamente). El acceso abierto al instrumento puede facilitar que investigadores de distintas regiones de Brasil lo utilicen en sus respectivos contextos.

## Conclusión

Con base en los resultados obtenidos, se concluye que el instrumento presenta buena adecuación y consistencia interna, lo que indica que es confiable para medir los constructos propuestos. Tras la evaluación por expertos en la temática y por el público objetivo del instrumento, la versión final quedó compuesta por 11 preguntas con escala de respuesta tipo Likert y dos preguntas abiertas. El presente estudio resultó en un instrumento de autocompletado, fácil de utilizar y susceptible de adaptaciones para comprender las barreras y facilitadores en la prevención de la violencia autoinfligida en distintos contextos de Brasil. Estos resultados indican la relevancia y la solidez del instrumento para futuras investigaciones, estrategias de prevención y políticas de salud.
